# Lung ultrasonography to assess efficacy of intranasal and parenteral vaccinations for bovine respiratory disease (BRD) in dairy calves

**DOI:** 10.1371/journal.pone.0349953

**Published:** 2026-05-28

**Authors:** Enrico Fiore, Giorgia Taio, Nicola Morandi, Elisa Mazzotta, Matteo Gianesella, Ortensio Bonato, Arnaldo Azzolin, Anastasia Lisuzzo

**Affiliations:** 1 Department of Animal Medicine, Production and Health (MAPS), University of Padua, Legnaro, PD, Italy; 2 Boehringer Ingelheim Italia, Milano, MI, Italy; 3 Istituto Zooprofilattico Sperimentale delle Venezie, Legnaro, PD, Italy; 4 Independent Researcher, Vicenza, VI, Italy; Cornell University, UNITED STATES OF AMERICA

## Abstract

Vaccination is used to control bovine respiratory disease (BRD). The aim of this study was to evaluate BRD vaccine efficacy through the lung lesions area in dairy calves, also discriminating the lung health status at vaccine administration. One hundred forty-nine dairy calves were enrolled and divided according to vaccine protocol and initial lung condition: healthy (H-CTR; n = 17) and diseased (D-CTR; n = 24) control group; healthy (H-INT; n = 28) and diseased (D-INT; n = 18) intranasal group, healthy (H-VAC; n = 31) and diseased (D-VAC; n = 31) intranasal and parenteral group. Intranasal vaccination was against bovine parainfluenza-3 and respiratory syncytial viruses, while parenteral vaccination was also against *Mannheimia haemolytica.* Animals were assessed by clinical and ultrasonographical examinations at 10, 17, 38, 52 days; ultrasonography and lung lesion scores (US and LLS), and total consolidation area (TC_A) were greater in CTR with increasing levels over time in both H-CTR (US: 1.45 at 10d vs. 3.8–4.4 thereafter; LLS: 3.5 at 10d vs. 12.4–13.3 thereafter; TC_A: 3.9 cm^2^ at 10d vs. 9.8–15.4 cm^2^ thereafter) and D-CTR groups (LLS: 12.1 at 10d vs. 13.0–15.7 thereafter; TC_A: 23.4 cm^2^ at 10d vs. 34.9–49.4 cm^2^ thereafter). However, TC_A was maintained in H-INT and reduced in D-INT (22.3 cm^2^ at 10d vs. 14.3 cm^2^at 52d). The VAC had the lowest values in US, LLS and TC_A. The H-VAC preserved the initial condition for these parameters, while D-VAC showed a reduction in US (3.8 at 10d vs. 3.0 at 52d) and TC_A (18.4 cm^2^ at 10d vs. 10.9 cm^2^ at 52d). In conclusion, the combination of intranasal and parenteral vaccination reduced the progression of lung TC_A severity in both initially healthy and diseased female dairy calves.

## 1. Introduction

Bovine respiratory disease (BRD) complex is the most important cause of morbidity and mortality in beef and dairy cattle [1]. The BRD is a syndrome involving infectious agents, the host immune system, and environmental factors. Infectious agents include viruses, such as bovine herpesvirus type 1 (BHV-1), parainfluenza-3 (PI3V), bovine viral diarrhea virus (BVDV), and bovine respiratory syncytial virus (BRSV), and/or bacteria, such as *Mannheimia haemolytica*, *Mycoplasma bovis*, *Pasteurella multocida*, and *Histophilus somni.* Usually, infection with viruses increases the susceptibility of animals to bacterial diseases, although some bacteria can cause disease on their own [2,3]. BRD represents a major health issue in pre-weaned calves and can lead to high economic losses. Indeed, the neonatal period is a time of significant risk for BRD due to an immature immune system compared to adults [4].

Vaccination is a common practice to reduce the incidence of BRD and its detrimental effects in pre-weaned dairy calves [1,5]. In fact, vaccination is generally used to prevent the expression of clinical signs of disease following infection or to prevent the transmission of infection in the first place. When adequate numbers of animals also achieve immunity through vaccination then herd immunity is established. An inadequate herd immunity increases the risk of age-associated diseases, such as BRD in calves, with the spread and persistence of disease-associated pathogens [6–8]. Usually, intranasal vaccination is the first type of vaccine administered due to its ability to establish protective immunity in newborn calves in presence of maternal antibodies [4]. The resulting mucosal immunity prevents infection due to its action on the upper respiratory tract, and it generally provides short-lived protection [9,10]. Parenteral vaccination induces a systemic immunity against BRD pathogens and it is usually provided later in life due to potentially adverse effect of maternal antibodies hindering seroconversion, although it can still generate an active immune response during this condition [1]. However, exposure of calves to pathogens’ antigens through vaccination in the presence of maternal antibodies may generate an active immune response developing a clinical protection against pathogens [11]. This route of administration generally provides effective protection of deeper organs as the lungs [4,12].

Regular screening for BRD in pre-weaned dairy calves is not routinely performed on the farm and is usually based on the systemic signs and respiratory signs that characterize the clinical examination [13,14]. Furthermore, the correlation between clinical signs and lung lesions at slaughter is usually low, in contrast to lung ultrasonography, and may be influenced by the severity of disease, the time of screening, and the duration of disease [14]. Lung ultrasonography can easily discriminate between a healthy and abnormal lung condition [3,15]. The most common lesions that can be revealed during lung ultrasonography are comet tails, hepatization, fluid alveologram or bronchogram, and pleuritis [16]. Moreover, ultrasonography is a non-invasive, cost-effective and portable method to investigate in real-time different lesions in lungs [16]. Several types of lung ultrasonography scoring were applied, especially focusing on the type and depth of lesions [17]. However, a description of the lesions area in the lungs without categorization based on a cut-off in pre-weaned calves is lacking to the best of the authors’ knowledge. Nevertheless, lung ultrasonography shows in general a higher sensitivity (94%; 95% confidence interval: 69–100%) and specificity (100%; 95% confidence interval: 64–100%) than clinical scoring to detect lung lesions (sensitivity 30–72%; specificity 86–94%) [18–20]. These two parameters are important for the early detection of diseased animals, and for an accurate assessment of the health status of animals in order to avoid unnecessary antimicrobial treatment [3,5].

For these reasons, the objective of this study was to evaluate BRD vaccine efficacy through the lung lesions area in dairy calves, also discriminating between healthy and lesioned lungs at vaccine administration.

## 2. Materials and methods

### 2.1. Animals and study design

Animal care and procedures are in accordance with the Guide for the Care and Use of Laboratory Animals and Directive 2010/63/EU for animal experiments (National law: D.L. 26/2014). No invasive medical procedures outside the routine farm procedures were performed in this study. The study was performed with the written consent of each animal’s owner during all procedures.

This study is part of a larger project partially covered in the study by Lisuzzo et al. [21], with some similar procedures even though performed on other animals. Three dairy farms in Veneto region (Italy) positive to *Mannheimia haemolytica* among BRD pathogens on deep nasal swab and necropsy examination during the previous two years was involved in the study. All female Holstein-Friesian dairy calves born during the study period (November-February 2023) were evaluated. Animals were dried and moved to individual outdoor elevated calf hutches immediately after calving, and received 10% of the body weight (BW) of colostrum within 4 h after the calving. The used colostrum derived by a colostrum bank that breeder created by own cows. Only colostrum with >22% of BRIX was included in the colostrum bank.

Each farm received a vaccine protocol forming the following three groups: Group **CTR** (control group without vaccination; n = 67); Group **INT** (intranasal vaccination; n = 65); and Group **VAC** (intranasal and parenteral vaccination; n = 74). This group choice was established to prevent the potential bias effect of herd immunity considering that the co-presence of vaccinated and unvaccinated animals may have an effect in reducing the pathogens’ circulation and their excretion, leading to a protective effect in unvaccinated co-housing animals [22]. A longitudinal study design was then used for this study. All animals were evaluated with clinical examinations and lung ultrasound evaluations at four time points: T0 (10 ± 2 days of life), T1 (17 ± 2 d), T2 (38 ± 2 d), and T3 (52 ± 2 d). Each evaluation was performed by one veterinarian of the Department of Animal Medicine, Production, and Health (MAPS), University of Padua.

Immediately after the examination at T0, Groups INT and VAC received the intranasal vaccination (Bovalto ® Respi Intranasal; Boehringer Ingelheim Animal Health Italia S.p.A., Noventa Padovana, Italy) against modified-live PI3V and BRSV. After 1 week (T1), the Group VAC received the first parenteral vaccination (Bovalto® Respi 3; Boehringer Ingelheim Animal Health Italia S.p.A., Noventa Padovana, Italy) against inactivated PI3V, BRSV, and *Mannheimia haemolytica* to differentiate the administration time between the two different types of vaccines. Furthermore, the Group VAC received the parenteral vaccination booster after 3 weeks from T1 (T2) according to the vaccine manufacturer’s instructions.

Three different operators were involved during the trial on the farm: one operator performed clinical evaluations at all time-points; one operator performed ultrasound examination of all animals at all time-points; and one operator was responsible for vaccine administration. Furthermore, the operators who performed the clinical and ultrasound evaluations were not aware of the protocol applied on the farm.

### 2.2. Clinical examination and lung ultrasonography evaluation

The clinical examination included the respiratory score (RS) calculated on the basis of respiratory and clinical signs (i. cough; ii. nasal discharge; iii. ocular discharge and ear drop; and iv. rectal temperature) as described by McGuirk & Peek [14]. The RS considered animals as positive for clinical respiratory disease when the sum of the scores of the four parameters was ≥ 5, or if two parameters showed a score of at least 2.

Ultrasonography evaluations on the lungs were performed with a portable ultrasound scanner (Draminski Blue; Draminski® S.A., Olsztyn, Poland) equipped with a multi-frequency linear probe (L40/10 MHz, 6.0–15.0 MHz; Draminski® S.A., Olsztyn, Poland). All scans were performed with constant ultrasound settings frequency of 6.0 MHz, 15 cm depth acoustics window, 100% grey scale gain, and time-gain compensation was in a neutral position. Images were stored in DICOM (digital imaging and communications in medicine) format.

An ultrasonography score (US) on a 6-point scale was established in the field according to Ollivett & Buczinski [23]. Specifically, US = 0 stands for normal tissue; US = 1 stands for comet tail; US = 2 stands for lobular consolidation; US = 3 stands for one lobar consolidation; US = 4 stands for two lobar consolidations; US = 5 stands for three or more lobar consolidations. Based on US, animals were considered as healthy (US < 3) or affected by BRD (US ≥ 3). In addition, the lung ultrasound was performed in six areas: between the 10^th^ and 7^th^ intercostal spaces (ICS) for the caudal region; between the 6^th^ and 5^th^ ICS for the middle region; and between the 4^th^ and 2^nd^ ICS for the cranial region of both sides. The lung lesions identified in the six regions were converted to a numeric scale as follows: 0 − healthy lung; 1 − presence of comet tails; 2 − spot of lobular consolidation; 3 − lobar consolidation; 4 − lobar consolidation and comet tails; 5 − fluid alveologram/bronchogram; 6 − fluid alveologram/bronchogram and comet tails; 8 − lobar consolidation and fluid alveologram/bronchogram; 9 − lobar consolidation, fluid alveologram/bronchogram, and comet tails; and 11 − pleuritis. The score of the six investigated regions was summed to obtain a lung lesion score (LLS) [24].

After sampling, the total consolidation area (TC_A) expressed in cm^2^ was measured by summing the consolidation of the six investigated regions using Image-J software (Wayne Rasband, Rockville Pike, Bethesda, USA). This post-sampling evaluation was performed by only one different operator not involved in clinical or ultrasound examinations, or vaccination administration, with the only information of animal ID and time point evaluation.

Only animals that respected the inclusion criteria were used for the following statistical analysis: *i)* born by eutocic calving; *ii)* received 10% BW of colostrum within 4 h after calving; *iii)* no antibiotics administration against BRD pathogens, anti-inflammatory or corticosteroids treatments during the trial; and *iv)* completed all time-points of the study design.

Based on US at T0, animals were considered as healthy (US < 3) or affected by BRD (US ≥ 3). Consequently, the groups enrolled: healthy (**H-CTR**) and diseased (**D-CTR**) control groups (**CTR**); healthy (**H-INT**) and diseased (**D-INT**) intranasal group (**INT**), healthy (**H-VAC**) and diseased (**D-VAC**) intranasal and parenteral group (**VAC**).

Approximately 20% of the animals in each group identified as diseased according to the US were randomly selected to perform a deep nasal swab, which was subsequently subjected to bacteriological and virological examinations at the laboratory of Istituto Zooprofilattico Sperimentale delle Venezie (IZSVe; Legnaro, Padua, Italy).

### 2.3. Statistical analysis

Sample size was evaluated a priori by the G*power software ver. 3.1.9.7. The parameter used as discriminant was the total lung consolidation area to discriminate between diseased and healthy veal calves according to Lisuzzo et al. [24]. The total lung consolidation of healthy and diseased animals of the mentioned study was 1.25 and 30.1 cm^2^, respectively, with a standard deviation of 1.55. The α was set at 0.05 and β was set at 0.95. The results evidenced an effect size of 9.21 and a total sample size of 4 animals. Considering the vaccination effect applied in this study, a post-hoc power analysis was also performed on the results to ensure a power equal or greater than 0.80.

The statistical analysis was performed with R software ver. 4.2.3 (R Core Team, Vienna, Austria) with the Rcmdr package. The data distribution was normal considering the Shapiro-Wilk test. Consequently, a generalized linear repeated mixed model with gaussian family was applied using the fixed effect of group, time, lung health status at T0 according to US (healthy: US < 3; diseased: US ≥ 3) nested within group, and their interaction, and the random and repeated effect of the animal to assess the differences in RS, US, LLS, and TC_A. The hypotheses of linear model on the residuals were graphically assessed to evaluate homoscedasticity. A post-hoc pairwise comparison among least square means was performed using Tukey correction. A p-value ≤ 0.05 was used to consider statistically significant differences.

## 3. Results

### 3.1. Groups’ size

The Group CTR, INT and VAC included 67, 65, and 74 animals, respectively. From originally enrolled animals, 43 were excluded due to treatments (19 in CTR; 16 in INT; and 8 in VAC) and other 14 animals were excluded due to lack of at least 1 time-point evaluation (7 in CTR; 3 in INT; and 4 in VAC). Consequently, the groups CTR, INT and VAC were composed by 41, 46, and 62 animals. According to US at T0, the sub-groups enrolled: H-CTR, n = 17; D-CTR, n = 24; H-INT, n = 28; D-INT, n = 18; H-VAC, n = 31; and D-VAC; n = 31.

### 3.2. Deep nasal swabs findings

Bacteriological and virologic analysis revealed the presence and circulation of *Mannheimia haemolytica* in all groups. The presence and circulation of *Pasteurella multocida* was identified in the INT group at the end of the trial, whereas Coronavirus was evidenced in the VAC group.

### 3.3. Effect of vaccination according to initial lung health status

**Fig 1:**
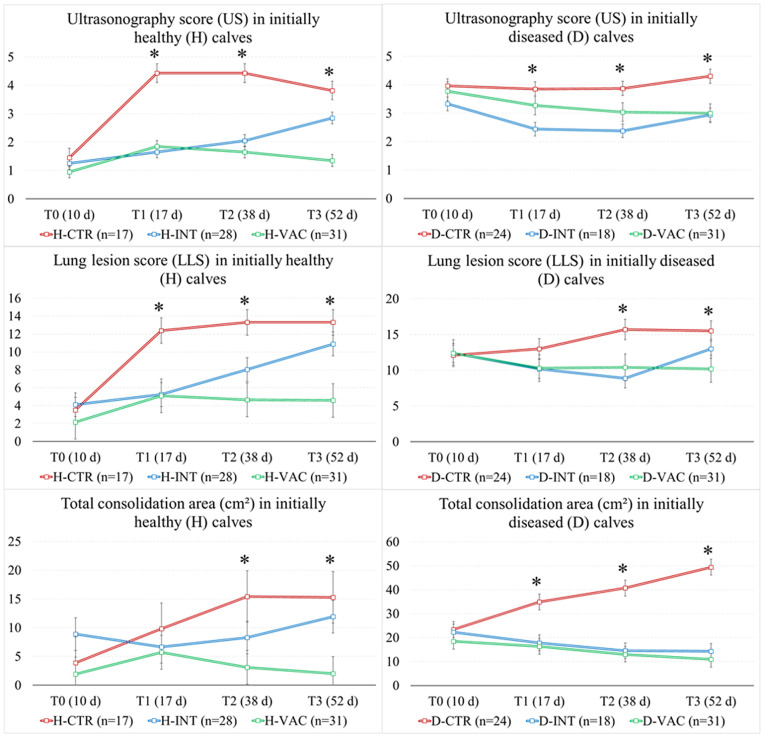
Significant parameters affected by lung health status at T0 (Healthy or H – US < 3; and Diseased or D – US ≥ 3) and to group (control group – CTR, n = 41; group with intranasal vaccination – INT, n = 46; group with intranasal and parenteral vaccinations – VAC, n = 62). (*) Indicated time points with a significant difference among groups. Specific information was provided in S1 Table.

The interaction among group, time and initial lung health status was significant for US (p = 0.004), LLS (p = 0.021), and TC_A (p = 0.026), while RS was not significant (p = 0.230). The D-CTR group had a stable US mean condition over time (3.85 to 4.30), while the H-CTR worsened over time (3.82–4.43 T1-T3 *vs.* 1.45 T0). The H-INT group showed an increase in US at the end of the study (2.85 T3 *vs.* 1.25–2.05 T0-T2), while the D-INT improved at 17 and 38 d (2.38–2.44 T1-T2 *vs.* 3.33 T0) with a slight increase at the end of the trial (2.94 T3). In contrast, the H-VAC group presented an increase in US only at 17 d (1.85 T1) and then returned to the initial values, while the D-VAC had a progressive improvement over time (3.0 T3 *vs.* 3.77 T0). Similar results were also found for the LLS. In contrast, the TC_A showed some differences. In fact, both H-CTR and D-CTR groups showed marked worsening over time (H-CTR: 15.3 T3 *vs.* 3.89 T0 cm^2^; D-CTR: 49.4 T3 *vs.* 23.4 T0 cm^2^). The H-INT group did not show any differences in TC_A over time (6.67 to 11.9 cm^2^) despite the slight increase in US and LLS at the end of the trial. In contrast, the D-INT had a progressive improvement with reduction in TC_A (14.3 T3 vs. 22.3 T0 cm^2^) despite the slight final increase in US and LLS. The H-VAC group presented no changes (1.93–5.71 cm^2^) despite the slight increase in US and LLS at 17d, while D-VAC had a progressive reduction in TC_A (10.9 T3 vs. 18.4 T0 cm^2^) (Fig 1; S1 Table). The median, first to thirds quartiles, minimum and maximum values of scores (RS, US, and LLS) were presented in Table 1.

**Table 1:** Median (First to Third quartile; Minimum to Maximum) of the respiratory score (RS), Ultrasonography score (US), and Lung lesion score (LLS) according to Group (control group – CTR; group with intranasal vaccination – INT; group with intranasal and parenteral vaccinations – VAC), Time (T0; T1; T2; and T3), and identified as healthy (H) or diseased (D) at T0 according to US (healthy – US < 3; and Diseased – US ≥ 3).

Parameter	Group	Status at T0	T0 (10 d)	T1 (17 d)	T2 (38 d)	T3 (52 d)
RS^1^	CTR (n = 41)	H-CTR (n = 17)	0.5 (0-1; 0-2)	0.5 (0-1; 0-2)	0 (0-1; 0-2)	1 (0-2; 0-2)
		D-CTR (n = 24)	1 (0-1.25; 0-3)	1 (1-2; 0-3)	1 (1-2; 0-2)	1 (0-2; 0-3)
	INT (n = 46)	H-INT (n = 28)	2 (1-2; 0-8)	1 (1-2; 0-5)	2 (1-2; 0-4)	2 (1-3; 0-5)
		D-INT (n = 18)	2 (1-2; 0-4)	1.5 (0-2; 0-3)	2 (1-2.5; 1-3)	2 (1 –3; 1 –4)
	VAC (n = 62)	H-VAC (n = 31)	1 (0-2; 0-3)	2 (1-2; 0-4)	1.5 (1-2; 0-4)	2 (2-3; 0-4)
		D-VAC (n = 31)	2 (1-2; 0-3)	2 (1-2; 0-3)	2 (2 –2; 2 –4)	1 (1-1.75; 0-2)
US^2^	CTR (n = 41)	H-CTR (n = 17)	2 (0.75-2; 0-2)	4.5 (4 –5; 3 –5)	4.5 (4 –5; 3 –5)	4 (3-4.5; 2-5)
		D-CTR (n = 24)	4 (3 –5; 3 –5)	4 (3 –5; 2 –5)	4 (3-4.75; 2-5)	4 (3 –5; 3 –5)
	INT (n = 46)	H-INT (n = 28)	1 (1-1.25; 0-2)	2 (1-2; 0-3)	2 (1.75-3; 0-5)	3 (2 –3; 2 –4)
		D-INT (n = 18)	3 (3-3.75; 3-5)	2 (2 –3; 1 –5)	2 (2-2.50; 2-4)	3 (2.5-3; 1-5)
	VAC (n = 62)	H-VAC (n = 31)	1 (0-1.25; 0-2)	2 (1-2; 0-4)	1.5 (1 –2; 1 –3)	1.5 (0-2; 0-4)
		D-VAC (n = 31)	4 (3 –4; 3 –5)	3 (3-3.25; 2-5)	3 (2 –4; 2 –4)	3 (2 –4; 2 –4)
LLS^3^	CTR (n = 41)	H-CTR (n = 17)	4 (2-5; 0-8)	12.5 (10-13.5; 7-25)	13 (10-15.25; 7-23)	13 (11.5-16.5; 9-22)
		D-CTR (n = 24)	12 (8.25-16; 3-24)	13 (11-15.5; 6-36)	16 (11 –19; 7-27)	16 (12 –20; 10 –22)
	INT (n = 46)	H-INT (n = 28)	3 (0-8; 0-12)	5 (2-8; 0-16)	7.5 (4.5-11; 0-14)	10.5 (8 –12; 4 –14)
		D-INT (n = 18)	12 (10 –13; 8-26)	10 (7 –12; 4 –19)	8.5 (6.75-11.25; 4-15)	13 (9 –14; 1 –15)
	VAC (n = 62)	H-VAC (n = 31)	2 (0-4; 0-10)	5 (2.75-6.5; 0-14)	4.5 (2.75-5.25; 1-9)	4 (2-7; 0-10)
		D-VAC (n = 31)	12 (4 –13; 5 –19)	10 (8.75-12.5; 5-14)	10 (7.5-12.5; 2-14)	10 (6.5-11; 2-12)
Total Consolidation area (cm^2^)	CTR (n = 41)	H-CTR (n = 17)	4 (2.5-7.0; 0-10)	10.5 (7 –13; 5 –18)	15.25 (9.5-20; 5-24)	16 (12.5-19.75; 9-30)
		D-CTR (n = 24)	25 (15-37; 12.5-42)	35 (31.5-38.75; 27-48.3)	42 (23.7-47.5; 21-54)	49.5 (32.5-58; 26-63.5)
	INT (n = 46)	H-INT (n = 28)	9.5 (8.25-12; 0-15)	7 (4.5-11; 0-14)	8.75 (4.25-14; 0-18)	12.25 (8.25-14.75; 0-21)
		D-INT (n = 18)	23.5 (18-26.5; 15-39)	18.5 (14.75-20; 6-33)	14.5 (8-19; 2.25-35)	14 (11 –18; 5-25)
	VAC (n = 62)	H-VAC (n = 31)	2 (1-4; 0-7)	5.5 (3-7; 0-12.25)	3.0 (1.5-3.2; 0-15.25)	2 (1-3.5; 0-15.5)
		D-VAC (n = 31)	18 (16-24.5; 6.5-31.4)	17 (10-23; 5-26.25)	14 (7.5-18; 0-23.25)	10.5 (5-15; 0-26)

^1^Respiratory score; ^2^ Ultrasonography score; ^3^ Lung lesion score.

### 3.4. General effect of vaccination

The CTR group showed lower values of RS compared to vaccinated groups (effect group, p = 0.001). However, no interaction between group and time was identified (group*time, p = 0.133). In contrast, both US and LLS (p = 0.001 for both) showed lower values in vaccinated animals (INT: 1.95 to 2.84 US, 7.19 to 11.57 LLS; VAC: 1.90 to 2.31 US, 6.77 to 7.86 LLS) compared to CTR group (3.04 to 4.22 US, 9.26 to 15.42 LLS). Moreover, the CTR group showed a worsening condition with an increase in US and LLS over time. Also, the INT group showed a worsening condition of both scores (US and LLS) at the end of the study period (T3, 52d). Despite this, the VAC group did not show any differences over time (Table 2).

**Table 2:** Main scores (RS, US, LLS) assessed in control group (CTR, n = 41), group with intranasal vaccination (INT, n = 46), and group with intranasal and parenteral vaccinations (VAC, n = 62) over time. Data were presented as mean (median; first to third quartile; minimum to maximum values).

Scores	Group	T0 (10 d)	T1 (17 d)	T2 (38 d)	T3 (52 d)	SEM	*p-value* ^*^
RS^1^	CTR	0.91^y^ (1; 0-1; 0-3)	1.07^y^ (1; 1-1; 0-3)	0.98^y^ (1; 0-1.75; 0-2)	1.20^y^ (1; 0-2; 0-3)	0.24	0.133
	INT	1.80^x^ (2; 1-2; 0-8)	1.39^xy^ (1; 0.5-2; 0-5)	1.69^x^ (2; 1-2; 0-4)	2.19^x^ (2; 1-3; 0-5)	0.19	
	VAC	1.14^xy^ (1; 0-2; 0-3)	1.72^x^ (2; 1-2; 0-4)	1.71^x^ (2; 1-2; 0-4)	1.97^x^ (2; 1-2; 0-4)	0.21	
US^2^	CTR	3.04^b^ (3; 2.75-4; 0-5)	4.06^x,a^ (4; 3 –5; 2 –5)	4.22^x,a^ (4; 3.25-5; 2-5)	4.21^x,a^ (4; 3 –5; 2 –5)	0.24	0.001
	INT	2.07^b^ (2; 1-3; 0-5)	1.95^y,b^ (2; 2-2.5; 0-5)	2.12^y,ab^ (2; 2-3; 0-5)	2.84^y,a^ (3; 2 –3; 1 –5)	0.19	
	VAC	2.31 (2; 1-3; 0-5)	2.29^y^ (2; 2-3; 0-5)	2.12^y^ (2; 1-2.5; 1-4)	1.90^z^ (2; 0.5-2.5; 0-4)	0.23	
LLS^3^	CTR	9.26^b^ (9; 6.5-14.5; 0-24)	13.16^x,a^ (13; 10.5-15.5; 6-36)	15.29^x,a^ (15; 11.25-18.25; 7-27)	15.42^x,a^ (15; 11.5-19; 9-22)	1.26	0.001
	INT	7.37^b^ (7; 5-11.75; 0-26)	7.19^y,b^ (7; 4-10; 0-19)	7.96^y,b^ (8; 5.75-10; 0-15)	11.57^x,a^ (11; 8 –13; 1 –15)	1.00	
	VAC	7.34 (7; 1-10; 0-19)	7.86^y^ (7; 4-10.5; 0-14)	6.77^y^ (6; 2.5-9; 1-14)	6.96^y^ (7; 2-9; 0-12)	1.16	

^1^Respiratory score; ^2^ Ultrasonography score; ^3^ Lung lesion score; ^*^ P-value of the interaction between group and time; ^a-b^ Mean values in the same row which differ significantly; ^x-z^ Mean values in the same column which differ significantly.

The consolidation area of cranial (p = 0.007 for left lung and p < 0.001 for right lung) and middle (p = 0.047 for left lung and p = 0.049 for right lung) regions and TC_A (p = 0.001) showed a similar condition to the ultrasound scores (US and LLS) with lower values in the vaccinated groups (Total: 10.29 to 14.14 cm^2^ in INT, 5.51 to 10.52 cm^2^ in VAC) than in the CTR group (Total: 17.67 to 33.53 cm^2^). In addition, the CTR group showed a marked worsening with a significant increase in consolidation areas during the trial. In contrast, the VAC group presented no change over time both considering cranial and middle regions and total area. In contrast, the INT group presented an increase in the consolidation area of the right cranial region and a decrease in the left cranial region. The outcome of this opposing condition was a maintenance of the TC_A over time (Table 3).

**Table 3:** Areas of consolidation (cm^2^) in the cranial, middle, and caudal regions of the right and left lungs in control group (CTR, n = 41), in the group with intranasal vaccination (INT, n = 46), and in the group with intranasal and parenteral vaccinations (VAC, n = 62) over time.

Consolidation Area	Group	T0 (10 d)	T1 (17 d)	T2 (31–38 d)	T3 (45–52 d)	SEM	*p-value* ^*^
Total Consolidation	CTR	17.67^c^	25.42^x,b^	30.44^x,ab^	33.53^x,a^	2.90	0.001
	INT	14.14	11.98^y^	10.29^y^	12.93^y^	2.36	
	VAC	10.52	9.23^y^	6.82^y^	5.51^z^	2.79	
*Left Lung*							
Cranial region	CTR	5.73^b^	8.06^x,a^	8.95^x,a^	8.26^x,a^	0.83 0.88 0.53 0.98 0.76 0.41 3.36	0.007
	INT	5.66^a^	4.16^y,ab^	3.70^y,ab^	3.00^y,b^	0.82	
	VAC	3.56	3.86^y^	2.87^y^	2.50^y^	0.96	
Middle region	CTR	1.91^c^	4.18^x,b^	4.65^x,b^	6.38^x,a^	0.88	0.047
	INT	1.36	1.45^y^	1.82^y^	1.51^y^	0.64	
	VAC	1.76	0.58^y^	0.57^y^	0.56^y^	0.68	
Caudal region	CTR	1.06	0.96	1.45	1.18	0.44	0.147
	INT	0	0	0	0	0.34	
	VAC	0	0	0	0	0.37	
*Right Lung*							
Cranial region	CTR	5.97^b^	8.81^x,ab^	10.87^x,a^	10.66^x,a^	1.15	<0.001
	INT	5.29^ab^	5.04^y,ab^	4.67^y,b^	7.19^y,a^	0.82	
	VAC	3.22	3.57^y^	2.48^z^	1.56^z^	0.93	
Middle region	CTR	2.56^b^	2.92^x,b^	2.97^x,b^	5.81^x,a^	0.81	0.049
	INT	2.41	1.43^xy^	1.15^y^	1.47^y^	0.67	
	VAC	1.72	0.79^y^	0.60^y^	0.60^y^	0.67	
Caudal region	CTR	0.35	0.50	1.58	1.38	0.33	0.122
	INT	0	0	0	0	0.26	
	VAC	0.24	0.34	0.36	0.22	0.29	

* P-value of the interaction between group and time; ^a-c^ Mean values in the same row which differ significantly; ^x-z^ Mean values in the same column which differ significantly.

## 4. Discussion

Animals vaccination is used to achieve herd immunity in order to reduce the pathogens spread, pathogen shedding, and severe clinical disease [6]. In fact, the study by Zhang et al. [22] showed that vaccination influences the immune effect in vaccinated calves but also promotes indirect immune effects in co-housing and non-vaccinated calves. Consequently, the vaccination groups (CTR, INT, and VAC) in this study were located in three different dairy farms to limit the bias effect of herd-immunity that could have a protective effect for the unvaccinated group (CTR). However, this choice may be a limitation of the study because the potential bias on farm or management effect that may have increased. In order to limit the farm bias, the three dairy farms were selected in a similar in geographic area, herd size, and epidemiological conditions. Despite this selection, the bias effect of farm cannot be completely ruled out and this condition represents a limitation of this study when evaluating its results.

One factor to consider when interpreting the following results is the type of criterion used to define healthy or diseased calves using lung ultrasound. In fact, there are different types of assessment and thresholds based on the size and/or type of lung lesions. Furthermore, these elements are still under discussion as lung ultrasonography in cattle is relatively recent, with only about 15 years of research [19,20]. In this study, we based our findings on only one of these criteria, mentioned above, based on the study of Ollivett & Buczinski [23]. The BRD risk is greater during the neonatal period due to an immature immune system compared to adults [4]. Generally, vaccination is used to manage this risk reducing the BRD detrimental effects in pre-weaned dairy calves [1,5]. Clinical signs and respiratory score are often used for a diagnosis on the farm, but they are poorly associated with lungs lesions. In contrast, lung ultrasonography can easily discriminate between a healthy and abnormal lung condition for a better identification of diseased animals [24]. However, a description of the lung lesions area without categorization based on a cut-off in pre-weaned calves is lacking to the best of the authors’ knowledge.

The unvaccinated calves are reported to display severe clinical signs of BRD more frequently [5]. In the present study, several animals of all groups never showed a clinical score indicative of BRD. The clinical signs’ severity may be influenced by a variability in immune response to pathogen or ongoing subclinical BRD [2,25]. Typically, the first clinical sign is the increase in body temperature, followed by other signs as nasal and ocular discharges, and droopy ear [26]. However, a continuous daily measurement of body temperature was not used in this study, so the hyperthermia peak might not be evidenced; cough, and nasal and ocular discharges may also refer to a diseased of the upper respiratory tract, and not to the lower respiratory tract (lungs and pleura) [3]. Indeed, the correlation between clinical signs and gross lung lesions depends on several factors such as the severity of the disease, the time of screening, and the duration of disease [14,24]. In contrast, lung ultrasound showed a higher correlation with post-mortem findings of lung lesions [23,24,27].

A systematic lung ultrasonography can identify changes in lung health status supporting diagnosis and treatment decisions [24,27]. The consolidated lung represents non-aerated tissue, and therefore non-functional, due to acute or chronic inflammatory processes [3]. In addition, areas of consolidation can be found early in life in calves, already in 2-week-old calves [28]. In the present study, lung lesions were recorded at the beginning of the trial (10d; T0) in all groups as evidenced by US, LLS and consolidation areas. In addition, the CTR group showed a worsening status over time in US, LLS, and TC_A, and in both initially H-CTR and D-CTR animals. This result may be related to the spread of pathogens resulting in the formation of lung lesions with an ongoing active bronchopneumonia characterized by inflammation and infection [19,29]. In contrast to CTR group, vaccinated animals showed a general US and LLS under the disease threshold (US < 3, and LLS < 10.5 [23,24]) during the trial, and lower areas of consolidation.

Mucosal immunity from intranasal vaccination increases the prevention of infection [9]. Moreover, the mucosal immune response has been reported to be effective in dairy calves younger than 10 days old even in the presence of maternal antibodies [5]. However, this immunity generally provides short-term protection, usually less than 4 months [10,30]. The INT group presented an increase in US and LLS at the end of the trial (52d, T3) regardless of initial lung status. However, this increase was not indicative of disease for the US (mean value 2.84 at T3), and slightly above the threshold for LLS (mean value 11.57 at T3). This final worsening was consistent with a short protection [10], and could also be due to the circulation of pathogens toward which the animals did not receive vaccination (*Mannheimia haemolytica* and *Pasteurella multocida*). However, intranasal vaccination administered in the first week of life was reported to be effective in preventing lung lesions extension compared with unvaccinated calves [5] as well as in the present study. In fact, the INT group showed a lower TC_A compared to CTR group (10.3 to 14.1 cm^2^, SEM 2.36 for INT *vs.* 17.7 to 33.5 cm^2^, SEM 2.90 for CTR). In addition, animals in the H-INT group maintained the TC_A over time. In contrast, the D-INT showed a reduction of the same. Therefore, it is possible that performing intranasal vaccination provided initial protection against some viruses (PI3V and BRSV in this study) that promoted a better disease management by the animal than unvaccinated animals.

In contrast to intranasal vaccination, parenteral vaccination provides longer immunity but requires at least 2 doses to induce effective protection especially with inactivated pathogens [1]. The VAC group did not show changes in US, LLS, and TC_A over time by always being the lowest values in these parameters compared to the CTR group (US: 1.9 to 2.3, SEM 0.23, *vs.* 3.0 to 4.2, SEM 0.24; LLS: 6.8 to 7.9, SEM 1.16, *vs.* 9.3 to 15.4, SEM 1.26; TC_A: 5.5 to 10.5 cm^2^, SEM 2.79, *vs.* 17.7 to 33.5 cm^2^, SEM 2.90), and exclusively for 52d compared to the INT group. Distinguishing the initial lung status, the H-VAC group had an increase of US and LLS, always below the applied threshold values in this study for BRD affecting the lower respiratory tract, at 17d (T1) potentially due to the identification of Coronavirus cases toward which the vaccines did not provide protection. In fact, the area of lung consolidation was not consistently affected. Instead, the D-VAC animals showed a progressive reduction in US and TC_A. A reduction in areas of consolidation can be attributed to the process of tissue healing following active bronchopneumonia [3,24]. Consequently, intranasal and parenteral vaccinations were effective in reducing the severity of lung pathology observed by lung ultrasonography in initially healthy animals. Indeed, the same vaccination in initially diseased animals may have favoured the reduction of additional injuries due to the protective action of vaccination.

Therefore, this study evidenced that the vaccination of calf herds can influence the progression of BRD, affecting the lower respiratory tract. In particular, a combination of intranasal and parenteral vaccinations appears to reduce the severity of lung pathology by up to 52 days, according to the results of this study. Furthermore, it should be noted that periodic monitoring of farm epidemiological conditions may be an important factor in reviewing vaccination protocols. The specific environmental, management conditions and epidemiology effect of the farms may have influenced the results of this study. Therefore, these factors represent a limitation, and the results’ generalization should be approached with caution, particularly when environmental and management conditions differ from those reported in this trial. For this reason, further studies involving larger and more diversified animal populations are needed to validate the findings.

## 5. Conclusions

In conclusion, both intranasal and combined (intranasal and parenteral) vaccine protocols were effective in preserving lung health status monitored by lung ultrasonography in female dairy calves. Healthy animals that received only the intranasal protocol against PI3V and BRSV preserved the lung TC_A. The same vaccine protocol on diseased animals could have promoted a better management of disease by animals. Instead, the combined (intranasal against PI3V and BRSV, and parenteral against PI3V, BRSV and *Mannheimia haemolytica*) vaccine protocol was able to preserve the TC_A when administered in healthy animals, and improve the lung healing by reducing additional injuries of the disease when administered in these animals.

## Supporting information

S1 Table Main scores (RS, US, LLS) and total consolidation area (cm^2^) according lung health status at T0 (Healthy or H – US < 3; and Diseased or D – US ≥ 3) and to group (control group – CTR, n = 41; group with intranasal vaccination – INT, n = 46; group with intranasal and parenteral vaccinations – VAC, n = 62). (PDF)
