# Supplementary material for: Lung ultrasonography to assess efficacy of intranasal and parenteral vaccinations for bovine respiratory disease (BRD) in dairy calves
Source: PLoS One. 2026 May 28;21(5):e0349953. doi: 10.1371/journal.pone.0349953 (PMC13218469; doi:10.1371/journal.pone.0349953)
Supplement: S1 Table — (PDF) [file pone.0349953.s001.pdf]

Lung Ultrasonography to Assess Efficacy of Intranasal and Parenteral Vaccinations for Bovine Respiratory Disease (BRD) in Dairy Calves

Enrico Fiore[a], Giorgia Taio[a], Nicola Morandi[b], Elisa Mazzotta[c], Matteo Gianesella[a], Ortensio Bonato[d], Arnaldo Azzolin[d], and Anastasia Lisuzzo[a,*]

S1 Table. Main scores (RS, US, LLS) and total consolidation area (cm^2) according lung health status at T0 (Healthy or H – US<3; and Diseased or D - US≥3) and to group (control group – CTR, n=41; group with intranasal vaccination – INT, n=46; group with intranasal and parenteral vaccinations – VAC, n=62).

Parameters	Group	Status at T0	T0 (10 d)	T1 (17 d)	T2 (38 d)	T3 (52 d)	SEM	p-value[*]
RS[1]	CTR	H-CTR (n=17)	0.70	0.67	0.54	1.04	0.39	0.230
		D-CTR (n=24)	1.00	1.25	1.30	1.30	0.34	
	INT	H-INT (n=28)	1.96	1.51	1.79	2.21	0.28	
		D-INT (n=18)	1.56	1.22	1.57	2.16	0.31	
	VAC	H-VAC (n=31)	0.95	1.75	1.50	2.20	0.32	
		D-VAC (n=31)	1.56	1.63	2.31	1.15	0.28	
US[2]	CTR	H-CTR (n=17)	1.45 [y,b]	4.43 [w,a]	4.43 [x,a]	3.82 [x,a]	0.33	0.004
		D-CTR (n=24)	3.96 [x]	3.85 [w]	3.87 [x]	4.30 [x]	0.25	
	INT	H-INT (n=28)	1.25 [y,c]	1.65 [z,bc]	2.05 [z,b]	2.85 [y,a]	0.21	
		D-INT (n=18)	3.33 [x,a]	2.44 [y,b]	2.38 [yz,b]	2.94 [y,ab]	0.24	
	VAC	H-VAC (n=31)	0.95 [y,b]	1.85 [yz,a]	1.65 [z,ab]	1.35 [z,b]	0.21	
		D-VAC (n=31)	3.77 [x,a]	3.27 [x,ab]	3.04 [y,b]	3.00 [y,b]	0.33	
LLS[3]	CTR	H-CTR (n=17)	3.5 [y,b]	12.4 [x,a]	13.3 [x,a]	13.3 [x,a]	1.82	0.021
		D-CTR (n=24)	12.1 [x,b]	13.0 [x,ab]	15.7 [x,a]	15.5 [x,a]	1.41	
	INT	H-INT (n=28)	4.11 [y,b]	5.24 [y,b]	8.03 [y,a]	10.9 [xy,a]	1.16	
		D-INT (n=18)	12.4 [x,a]	10.2 [x,ab]	8.85 [y,b]	13.0 [x,a]	1.33	
	VAC	H-VAC (n=31)	2.15 [y,b]	5.1 [y,a]	4.65 [z,ab]	4.58 [z,ab]	1.18	
		D-VAC (n=31)	12.4 [x]	10.3 [x]	10.4 [y]	10.2 [y]	1.88	
Total Consolidation area (cm^2)	CTR	H-CTR (n=17)	3.89 [y,b]	9.81 [yz,ab]	15.4 [y,a]	15.3 [y,a]	4.48	0.026
		D-CTR (n=24)	23.4 [x,c]	34.9 [x,b]	40.7 [x,b]	49.4 [x,a]	3.31	
	INT	H-INT (n=28)	8.89 [y]	6.67 [z]	8.28 [yz]	11.9 [yz]	2.84	
		D-INT (n=18)	22.3 [x,a]	17.8 [y,ab]	14.5 [y,b]	14.3 [y,b]	3.28	
	VAC	H-VAC (n=31)	1.93 [y]	5.71 [z]	3.09 [z]	2.03 [z]	2.95	
		D-VAC (n=31)	18.4 [x,a]	16.3 [y,ab]	13.0 [y,ab]	10.9 [y,b]	3.24	

[1] Respiratory score; [2] Ultrasonography score; [3] Lung lesion score; [*] P-value of the overall interaction (group*time*status at T0); [a-c] Mean values in the same row which differ significantly; [w-z] Mean values in the same column which differ significantly.
